# Supplementary material for: Rapid COVID-19 Molecular Diagnostic System Using Virus Enrichment Platform
Source: Biosensors (Basel). 2021 Oct 6;11(10):373. doi: 10.3390/bios11100373 (PMC8534047; doi:10.3390/bios11100373)
Supplement: Supplementary file 1 [file biosensors-11-00373-s001.zip › biosensors-1390613-supplementary.pdf]

Supplementary Materials

Rapid COVID-19 Molecular Diagnostic System Using Virus Enrichment Platform

Yoon Ok Jang [1,†], Hyo Joo Lee [1,2,†], Bonhan Koo [1], Hye-Hee Cha [2], Ji-Soo Kwon [2], Ji Yeun Kim [2], Myoung Gyu Kim [1,3], Hyun Soo Kim [4], Sung-Han Kim [2,*] and Yong Shin [1,*]

[1] Department of Biotechnology, College of Life Science and Biotechnology, Yonsei University, Seoul 03722, Korea; jangyo17@daum.net (Y.O.J.); hyoj0125@gmail.com (H.J.L.); qhsgksdlek@naver.com (B.K.); wws94@naver.com (M.G.K.)
[2] Department of Infectious Diseases, Asan Medical Center, University of Ulsan College of Medicine, Songpa-gu, Seoul 05505, Korea; heyhe0102@naver.com (H.-H.C.); kwonjs92@kaist.ac.kr (J.-S.K.); aeki22@snu.ac.kr (J.Y.K.)
[3] Department of Convergence Medicine, Asan Medical Institute of Convergence Science and Technology (AMIST), University of Ulsan College of Medicine, Songpa-gu, Seoul 05505, Korea
[4] INFUSIONTECH, 38, Heungan-daero 427 beon-gil, Dongan-gu, Anyang-si 14059, Korea; hskim@infusiontech.co.kr
* Correspondence: kimsunghanmd@hotmail.com (S.-H.K.); shinyongno1@yonsei.ac.kr (Y.S.)
† These authors contributed equally to the work.

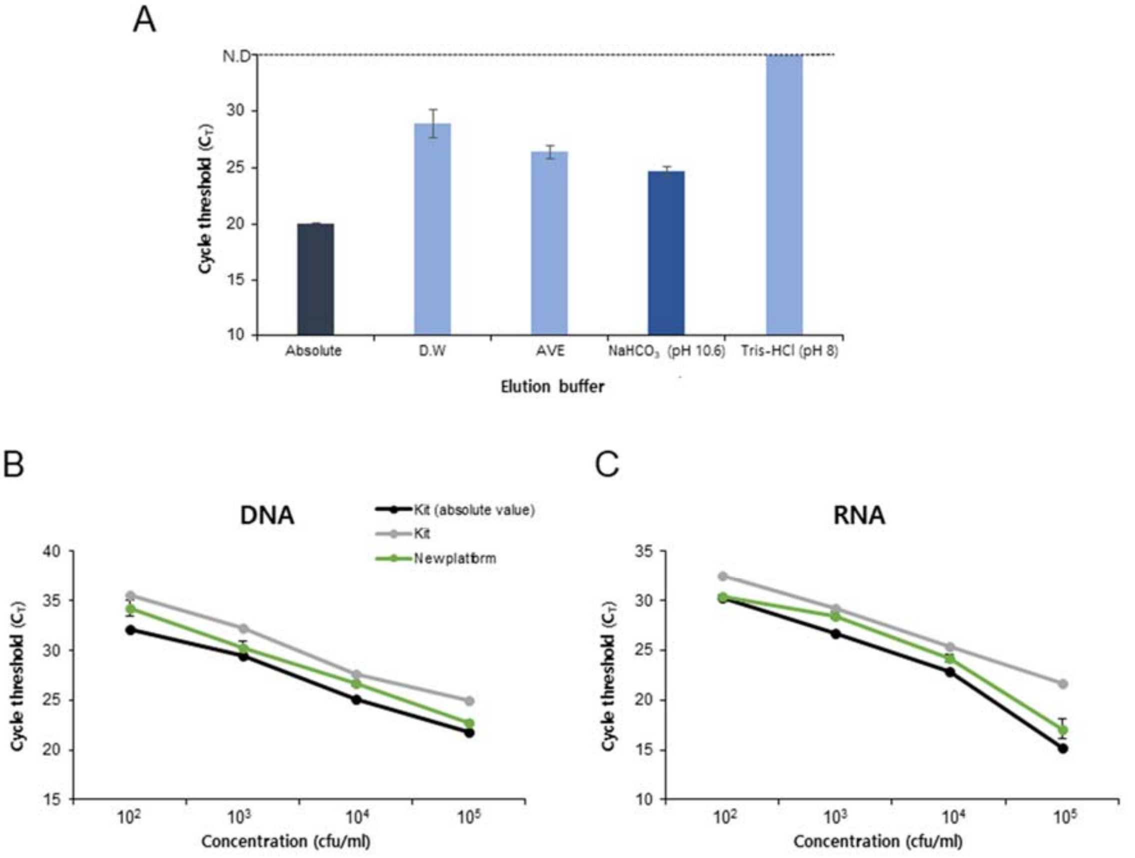

Figure S1. Application of DMP-PVDF filter platform for pathogen enrichment and extraction. (A) The efficiency of DMP-PVDF filter platform for pathogen extraction is dependent on the elution buffer. The positive control (gray) was DNA extracted from *E. coli* (10^5 CFU/mL) using a Qiagen kit. The highest amplification efficiency of the elution buffer on the DMP-PVDF filter platform was seen the 10 mM

NaHCO3 (pH 10.6, dark blue). (B and C) Concentration for (B) DNA or (C) RNA extraction to process test in concentrations ranging from 1 × 10^2 to 1 × 10^5 CFU/mL using both the NA extraction only without enrichment using a Qiagen kit (gray) and the new platform (light green). White color denotes NA extracted from the absolute concentration sample of using a Qiagen kit. The data are presented as mean ± SD, based on at least three independent experiments. DMP, dimethyl pimelimidate; PVDF, polyvinylidene fluoride; $NaHCO_3$, sodium bicarbonate; NA, nucleic acid; SD, standard deviation.

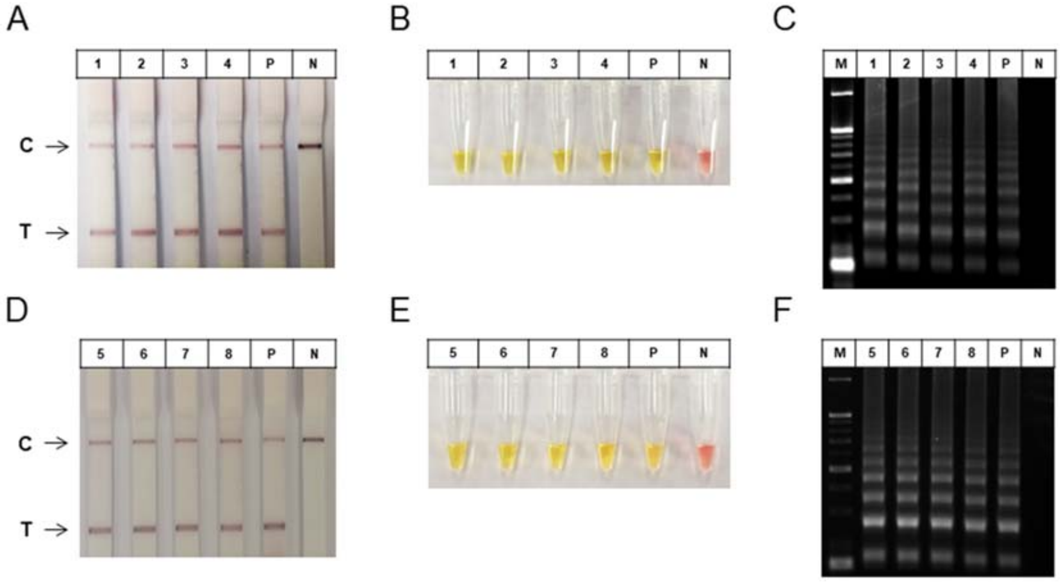

Figure S2. Optimization of RT-LAMP primers for SARS-CoV-2 detection. RT-LAMP was performed using synthetic RNA for S and N genes of SARS-CoV-2. (A-C) RT-LAMP with the S gene LAMP primers could detect the viral RNA (5.85 × 10^9 to 5.85 × 10^6 per reaction). (A) RT-LAMP with LFA, (B) colorimetric-LAMP, and (C) agarose gel electrophoresis of the reaction products. (D-F) RT-LAMP with the N gene LAMP primers could detect the viral RNA (2.33 × 10^9 to 2.33 × 10^6 per reaction). (D) RT-LAMP with LFA, (E) colorimetric-LAMP, and (F) agarose gel electrophoresis of reaction products. All RT-LAMP reactions were performed at 65 °C for 60 min. RT, reverse transcription; LAMP, loop-mediated isothermal amplification; S, spike; N, nucleocapsid; LFA, lateral flow assay; M, size marker; P, positive control; N; negative control; T, target; C, control.

Table S1. Primer sequences.

Primer	Sequences 5′-3′
E.coli-F	CAACGAACTGAACTGGCAGA
E.coli-R	CATTACGCTGCGAGGAT
Brucella-F	GCTTGAAGCTTGCGGACAGT
Brucella-R	GGCCTACCGCTGCGAAT
N gene T7-Fragment-F	TTTGGTGGACCCTCAGATTC
N gene T7-Fragment-R	TTGGCAATGTTGTTCCTTGA
S gene T7-Fragment-F	AGAACTCAATTACCCCCTGCAT
S gene T7-Fragment-R	CGAAAAACCCTGAGGGAGAT
N gene RT-qPCR-F	TGGCAGTAACCAGAATGGAGAAC

N gene RT-qPCR-R	AGTGAGAGCGGTGAACCAAGA
N gene RT-qPCR-P	FAM-CGCGATCAAAACAACGTCGGCC-BHQ_1
S gene RT-qPCR-F	TCCGCATCATTTTCCACTTTTAA
S gene RT-qPCR-R	AAGTCAGACAAATCGCTCCAGGGCAAA
S gene RT-qPCR-P	CY5-TAACGCAGCCTGTAAAATCATCTG-BHQ_2
N gene RPA-F	AATAAGCATATTGACGCATACAAAACATTC
N gene RPA-R	AGGCCTGAGTTGAGTCAGCACTGCTCATGGATTGT
N gene HDA-F	TCACTCAACATGGCAAGGAAGACCTTA
N gene HDA-R	CGAATTCGTCTGGTAGCTCTTCGGTAG
S gene LAMP-F3	TCTTTCACACGTGGTGTT
S gene LAMP-B3	GTACCAAAAATCCAGCCTC
S gene LAMP-FIP	CATGGAACCAAGTAACATTGGAAAATTTTCCTGACAAAGTTTTCAGATCC
S gene LAMP-BIP	CTCTGGGACCAATGGTACTAAGAGTTTTGACTTCTCAGTGGAAGCA
S gene LAMP-LF	GAAAGGTAAGAACAAGTCCTGAGT
S gene LAMP-LB	CCCTGTCCTACCATTTAATGATGG
N gene LAMP-F3	TGGCTACTACCGAAGAGCT
N gene LAMP-B3	TGCAGCATTGTTAGCAGGAT
N gene LAMP-FIP	TCTGGCCCAGTTCCTAGGTAGTTTTTGACGAATTCGTGGTGGTGA
N gene LAMP-BIP	AGACGGCATCATATGGGTTGCATTTTGCGGGTGCCAATGTGATC
N gene LAMP-LF	ACCATCTTGGACTGAGATCTTTCA
N gene LAMP-LB	GAGGGAGCCTTGAATACACCA

F, forward; R, reverse; P, probe; F3, forward outer primer; B3, backward outer primer; FIP, forward inner primer; BIP, backward inner primer; LF, loop forward primer; LB, loop backward primer; N, nucleocapsid; S, spike; RPA, recombinase-based polymerase amplification; HDA, helicase-dependent amplification; LAMP, loop-mediated isothermal amplification
